# Protective Effects of Metallothionein on Isoniazid and Rifampicin-Induced Hepatotoxicity in Mice

**DOI:** 10.1371/journal.pone.0072058

**Published:** 2013-08-13

**Authors:** Yong Lian, Jing Zhao, Peiyu Xu, Yimei Wang, Jun Zhao, Li Jia, Ze Fu, Li Jing, Gang Liu, Shuangqing Peng

**Affiliations:** 1 Evaluation and Research Centre for Toxicology, Institute of Disease Control and Prevention, Academy of Military Medical Sciences, Beijing, China; 2 Department of Toxicology, West China School of Public Health, Sichuan University, Chengdu, China; Temple University School of Medicine, United States of America

## Abstract

Isoniazid (INH) and Rifampicin (RFP) are widely used in the world for the treatment of tuberculosis, but the hepatotoxicity is a major concern during clinical therapy. Previous studies showed that these drugs induced oxidative stress in liver, and several antioxidants abated this effect. Metallothionein (MT), a member of cysteine-rich protein, has been proposed as a potent antioxidant. This study attempts to determine whether endogenous expression of MT protects against INH and RFP-induced hepatic oxidative stress in mice. Wild type (MT+/+) and MT-null (MT−/−) mice were treated intragastrically with INH (150 mg/kg), RFP (300 mg/kg), or the combination (150 mg/kg INH +300 mg/kg RFP) for 21 days. The results showed that MT−/− mice were more sensitive than MT+/+ mice to INH and RFP-induced hepatic injuries as evidenced by hepatic histopathological alterations, increased serum AST levels and liver index, and hepatic oxidative stress as evidenced by the increase of MDA production and the change of liver antioxidant status. Furthermore, INH increased the protein expression of hepatic CYP2E1 and INH/RFP (alone or in combination) decreased the expression of hepatic CYP1A2. These findings clearly demonstrate that basal MT provides protection against INH and RFP-induced toxicity in hepatocytes. The CYP2E1 and CYP1A2 were involved in the pathogenesis of INH and RFP-induced hepatotoxicity.

## Introduction

Tuberculosis (TB) is an airborne infectious disease and remains one of the major public health problems in the world today. More than 10 million people develop tuberculosis annually, and about 2 million die each year [1]. Isoniazid (INH) and Rifampicin (RFP) are first-line drugs for anti-TB therapy, but the hepatotoxicity that results from use of these drugs remains a significant problem for clinical treatment [2]. INH, the hydrazide of isonicotinic acid, is highly bactericidal against replicating tubercle bacilli. INH is directly or indirectly metabolized to acetylhydrazine and hydrazine by N-acetyltransferase and amidohydrolase [3]. Acetylhydrazine and hydrazine might be oxidized by CYPs to form hepatotoxic intermediates [4]. Human genetic studies have shown that CYP2E1 is involved in INH-related hepatotoxicity [5]. RFP is a complex macrocyclic antibiotic that inhibits ribonucleic acid synthesis in a broad range of microbial pathogens. It has bactericidal action and a potent sterilizing effect against tubercle bacilli in both cellular and extracellular locations. RFP is considered as a powerful inducer of mixed-function oxidase that contributes to the hepatotoxicity of INH [6]. Yuhas et al. showed rifampin can induce inflammatory mediators and enhance cytokine-induced production of NO and IL-8 in a liver epithelial cell line [7]. Various forms of CYP, such as CYP1A1, CYP1A2 and CYP2E1, are involved in free radical generation and RFP-mediated free radical generation may be associated with alterations in the expression of CYPs.

Previous studies have demonstrated oxidative stress in patients having anti-TB drugs induced hepatotoxicity [8]. Some studies show the fight against oxidative stress is likely to play a role in liver protection [9], [10]. Peroxidation of endogenous lipids has been shown to be a major factor in the cytotoxic action of INH and RFP [11]. The mechanism is generally attributed to the formation of the highly reactive oxygen species (ROS), which act as stimulators of lipid peroxidation and the source for destruction and damage to the cell membrane [12]. As far as we know, there are two antioxidant defense systems in the organisms: one is enzymatic, including superoxide dismutase (SOD), glutathione peroxidase (GPx), catalase (CAT), etc, the other one is non-enzymatic, which comprises molecules of low molecular weight that scavenges the free radicals to minimize the fluctuations of ROS level. Examples are glutathione (GSH) and metallothionein (MT). Recently, it has been found that INH induced cholestasis through enhancement of bile acid accumulation and mitochondria β-oxidation [13]. Moreover, alterations of various cellular defense mechanisms have been reported to be involved in INH and RFP-induced hepatotoxicity [14].

Metallothionein (MT) is a low-molecular mass (6–7 kDa), inducible, intracellular protein that is rich in cysteine (33% as cysteine residue). There are four isoforms of MTs, namely MT-I, MT-II, MT-III and MT-IV. MT-I and MT-II are widely expressed in all tissues, whereas MT-III and MT-IV are expressed mainly in the central nervous system and the squamous epithelia respectively [15]. MTs play an important role in many physiological processes including homeostasis, protection against heavy metals and oxidant damage, immune response, metabolic regulation, sequestration and/or redox control [16], [17]. In recent years, MT has also been proposed as an antioxidant based on its sulfhydryl-rich nature [18], [19]. Mechanisms underlying the potential antioxidant activity of MT include the direct scavenging of free radicals, altered Zn homeostasis, or interaction with glutathione (GSH).

It has been difficult to define the physiological function of MT using conventional animal models because the methods used to alter MT levels in animals also alter a wide variety of cellular reactions [20]. With the generation of mouse models that either overexpress [21] or do not express MT [22], [23], it is now possible to study more directly the role of MT in specific cellular processes. Liu et al. found MT deficiency renders animals more vulnerable to acetaminophen-induced hepatotoxicity [24]. Further, by using a MT-overexpressing transgenic mouse model, studies from Dr. Kang^’^s laboratory have demonstrated that acute alcohol hepatotoxicity and hepatic oxidative stress are significantly inhibited in MT-transgenic mice [25]. Our team recently revealed that MT-I/II null (MT−/−) mice are more sensitive than wild type (MT+/+) mice to butenolide-induced hepatic oxidative stress, indicating the antioxidant potency of basal MT [26]. Therefore, this study is designed to determine whether intracellular MT protects against INH and RFP-induced hepatic oxidative stress in mice.

## Materials and Methods

### Ethics Statement

All animal procedures were approved by the Institutional Animal Care and Use Committee at the Institute of Disease Control and Prevention of the Academy of Military Medical Sciences (2009-002).

### Chemicals and Antibodies

Isoniazid and rifampicin were purchased from Sigma-Aldrich Inc. (MO, USA). Goat anti-Cu-Zn superoxide dismutase (SOD-1) antibody, mouse anti-Mn superoxide dismutase (SOD-2) antibody, goat anti-CYP4501A2 antibody, donkey anti-goat IgG-HRP, goat anti-mouse IgG-HRP and goat anti-rabbit IgG-HRP were obtained from Santa Cruz Biotechnology Inc. (CA, USA). Rabbit anti-CYP4502E1 antibody was purchased from Abcam Ltd. (Hong Kong).

### Animals and Drug Treatment

Homozygous MT-I and -II knock-out mice (129/Ola×C57BL/6J background [22]) were obtained from the Murdoch Institute of the Royal Children’s Hospital (Parkville, Australia). Genetic background matched mice (129/Ola×C57BL/6J) were bred as controls. All the animals were kept in a ventilated animal room maintained at 23±2.5°C with a standard 12 hr/12 hr light/dark cycle, and given access to food and tap water *ad libitum*. Male 7–9-week-old mice were used for experimental studies.

Both wild-type and MT−/− mice were randomly assigned to four groups of six each, including a control group and three treatment groups. Mice in the treatment groups received 150 mg/kg of INH, 300 mg/kg of RFP or the combination of INH (150 mg/kg) and RFP (300 mg/kg), respectively. Control mice were administered with equivalent volumes of 1% carboxymethyl cellulose only. All animals were treated by intragastric administration (0.2 ml/10 g) daily for 21 consecutive days. INH and RFP were dissolved in 1% carboxymethyl cellulose. Animals were sacrificed on the next morning of the last administration. Blood was collected and livers were removed for further determination as described below.

### MT Concentration Assay

Hepatic MT concentrations were determined by a cadmium–hemoglobin affinity assay [27]. Briefly, liver tissues were homogenized in 9 volumes of 30 mM Tris-HCl buffer, pH 8.0. After centrifugation of the homogenate at 18,000×g for 15 min, supernatants were removed for MT determination by graphite furnace atomic absorption spectrometry.

### Plasma Biochemistry Analysis

Blood collected was allowed to clot at room temperature and then centrifuged at 3000 rpm for 10 min, and the resulting plasma was removed for biochemical assay within 2 hr. Typical parameters which are indicative of hepatic injury were determined: concentrations of alanine aminotransferase (ALT) and aspartate aminotransferase (AST).

### Liver Histopathology

Formalin-fixed livers were processed according to the routine procedure, and 5 µm of thickness was sectioned. The sections were stained with haematoxylin and eosin (H&E) for light microscopic examination.

### Lipid Peroxidation Assay

Lipid peroxidation was determined by measurement of malondialdehyde (MDA) formation using the thiobarbituric acid reactive substance assay as described previously [28].

### Determination of GSH Content and the Activities of GR, GPx, and SOD

The content of GSH was quantified following the method as previously reported by Beutler et al. using GSH as a standard [29]. Results were expressed as micromoles per milligram of protein. Glutathione reductase (GR) activity was assayed by measuring the rate of NADPH oxidation in the presence of GSSG [30]. Glutathione peroxidase (GPx) activity was assayed by measuring the rate of NADPH oxidation based on the reduction of peroxide hydroperoxide by GPx in the presence of GSH [31]. SOD activity was measured in liver homogenates using a commercialized kit (Nanjing Jiancheng Bioengineering Institute, PR China).

### Western Blot Analysis

Frozen portions of the livers were homogenized in liquid nitrogen and then lysed for 30 min on ice. The homogenized tissues were centrifuged thereafter at 4°C with 14,000×g for 25 min and supernatants were taken. Protein concentrations were determined and β-actin was used to normalize the protein loading. Protein (20 µg per lane) was separated on a 12% SDS-PAGE and electrophoretically transferred onto polyvinylidene fluoride (PVDF) membranes. After blocking, the membranes were processed for immunodetection with mouse anti-β-actin antibody, goat anti-SOD-1 antibody, mouse anti-SOD-2 antibody, goat anti-CYP4501A2 (NP034123.1) antibody and rabbit anti-CYP4502E1 (NP067257.1) antibody. The bound primary antibodies were detected with appropriate secondary antibody. The immunoreactive bands were visualized using the enhanced chemiluminescence method reagents (Amersham Pharmacia Biotech, NJ, USA) according to manufacturer's protocol and were quantitated by densitometric scanning of X-ray films (HP Scanjet G3010) and analyzed by the Quantity One 4.1.1 program (Bio-Rad).

### Statistical Analysis

Results were expressed as mean ± S.D. Statistical analysis of the data was determined by one-way ANOVA for multiple group comparisons. Differences between MT−/− mice and MT+/+ controls were analyzed by Student’s t test. A level of p<0.05 was considered significantly different.

## Results

### Hepatic MT Contents in MT−/− and MT+/+ Mice

**Figure 1:**
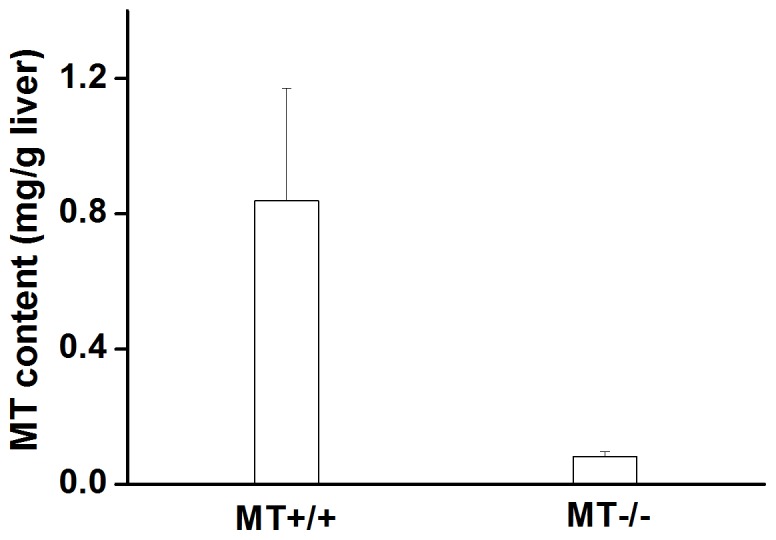
Hepatic MT concentrations in wild-type (MT+/+) and MT−/− mice. The MTconcentrations in the livers of MT+/+ and MT−/− mice were determined by cadmium-saturation assay. Data were expressed as means ± standard deviations.

To explore the potential antioxidant protection of MT, the present study employed MT-I/II null mice and the corresponding wild-type mice. As shown in Fig. 1, the basal concentration of hepatic MT in MT−/− mice was less than one-tenth that of MT+/+ mice. These results demonstrated the reliability of the animal models used in the study.

### Serum ALT, AST Concentration and Liver Index

As shown in Table 1, there were almost no significant alterations in the biochemical parameters following exposure to INH alone. Compared with control groups, RFP treatment produced remarkable increases in the concentrations of ALT, AST and liver index in both types of mice. Co-administration of RFP with INH dramatically increased the concentration of AST and liver index in MT−/− mice compared to control group.

**Table 1:** Effect of INH and RFP treatment on plasma ALT, AST activities and liver index of wild-type (MT+/+) and MT−/− mice.

Parameter		Control	INH	RFP	INH+RFP
ALT (U/L)	MT+/+	32.5±10.1	23.0±11.5	191.5±124.5**	53.0±27.5
	MT−/−	25.0±8.6	24.0±6.8	312.3±113.1**	75.0±50.2
AST (U/L)	MT+/+	150.5±48.3	130.5±29.2	266.0±130.8*	136±23.9
	MT−/−	135.5±28.3	160.5±37.0	284.0±90.3**	231.5±55.2** ##
Liverindex (%)	MT+/+	4.2±0.2	4.9±0.5*	6.6±0.4**	5.6±0.7**
	MT−/−	4.3±0.2	4.7±0.1	8.9±0.7** ##	7.0±0.5** ##

** p<0.01, compared with respective control;

* p<0.05, compared with respective control;

## p<0.01, compared with MT+/+ mice for the same treatment.

### Liver Histopathological Changes under Light Microscopy

**Figure 2:**
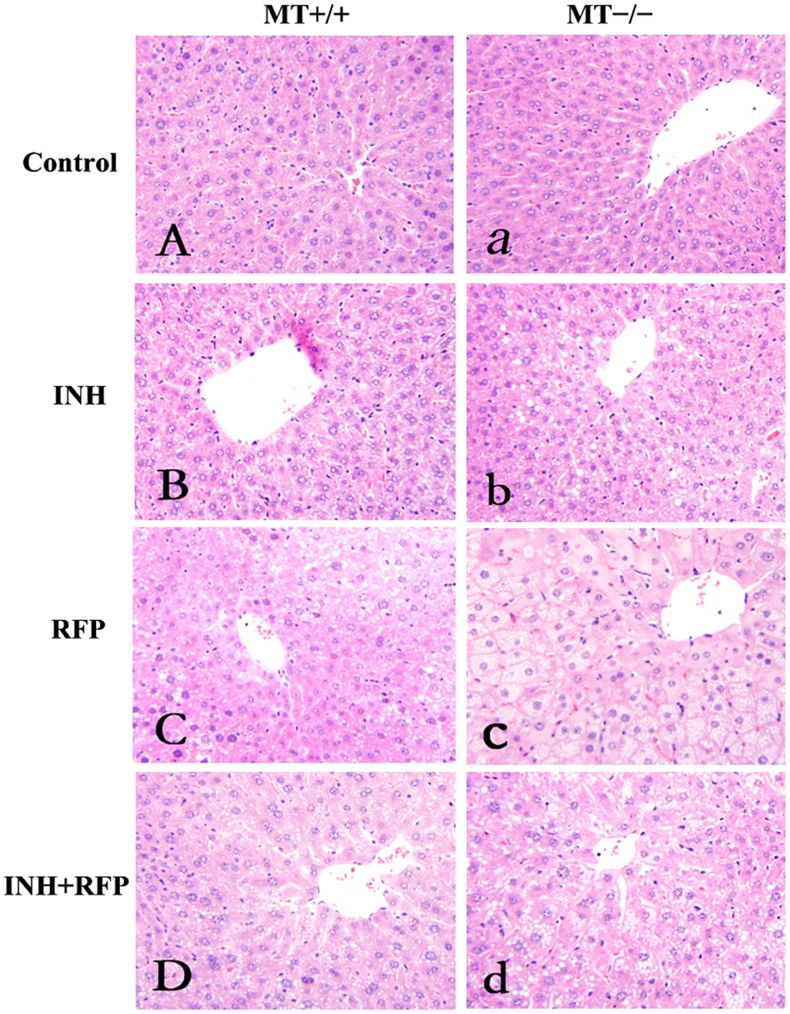
Liver histopathology of wild-type (MT+/+) and MT−/− mice treated with INH and RFP. INH/RFP (alone or in combination) induced apparent histopathological changes in both types of mice livers as exhibited by hepatocellular swelling, vacuolization, fatty degeneration, and these changes were more prominent in MT−/− mice. Livers from the MT+/+ mice and MT−/− mice were demonstrated in (A)–(D) and (a)–(d), respectively. H&E staining. Magnification, ×200.

Liver histopathologic examination was performed to further evaluate the hepatotoxicity of INH and RFP. As shown in Fig. 2, control livers showed no signs of obvious abnormality in both types of mice. After INH/RFP (alone or in combination) administration, apparent histopathological changes were observed in hearts from both types of mice, including hepatocellular swelling, vacuolization, fatty degeneration, and the alterations were more severe in MT−/− mice.

### Hepatic Lipid Peroxidation

Malondialdehyde, the well-known end product of lipid peroxidation, has been used as a biomarker of hepatic oxidative damage. As shown in Table 2, compared with the corresponding wild-type mice, the MDA level was markedly increased in the INH, RFP and combined group in MT−/− mice.

**Table 2:** Lipid peroxidation in livers of wild-type (MT+/+) and MT−/− mice treated with INH and RFP.

Parameter		Control	INH	RFP	INH+RFP
MDA (nmol/100 mg pro)	MT+/+	17.5±2.4	16.5±2.4	14.5±1.7	15.2±3.4
	MT−/−	17.4±4.0	23.8±3.2** ##	24.3±3.3** ##	18.9±4.4##

** p<0.01, compared with respective control;

## p<0.01, compared with MT+/+ mice for the same treatment.

### Antioxidant Status of Hepatic GSH, GPx, GR and SOD in Wild-type and MT−/− Mice

**Figure 3:**
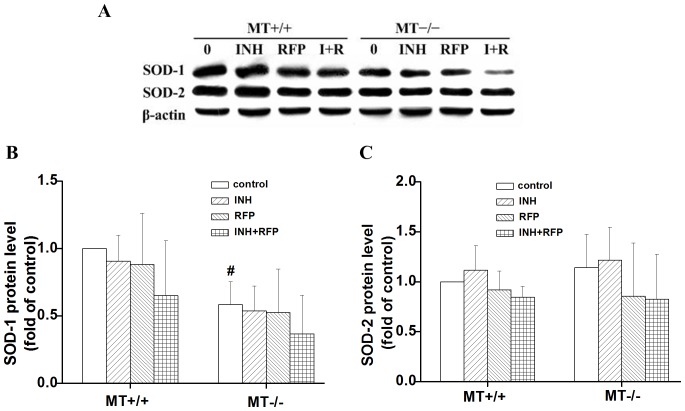
Effect of INH and RFP treatment on the expressions of SOD-1 and SOD-2 in wild-type (MT+/+) and MT−/− mice. The expression of SOD-1 and SOD-2 was analyzed by Western blotting. The control values of SOD-1 in MT−/− mice and MT+/+ were not identical, SOD-1 protein in MT−/− mice was significantly lower than that in wild-type mice. ^#^p<0.05, compared with MT+/+ mice for the same treatment.

GSH is one the important nonenzymatic antioxidants in the body. As presented in Table 3, RFP induced obvious increases in the content of hepatic GSH in both types of mice, but the percentages of GSH induction in MT−/− mice were higher than those in MT+/+ mice. Compared with the corresponding wild-type mice, the GSH was also markedly increased in the combined group in MT−/− mice. In all treatment groups, GPx activity showed a significant decrease compared to the control group. GR activity was strongly increased in the RFP and combined group, especially in the MT−/− mice. The control values of SOD in MT−/− and MT+/+ mice were not identical, and SOD-1 protein level in MT−/− mice was significantly lower than that in wild-type mice (Fig. 3).

**Table 3:** Effect of INH and RFP treatment on hepatic oxidative stress of wild-type (MT+/+) and MT−/− mice.

Parameter		Control	INH	RFP	INH+RFP
GSH (µmol/g pro)	MT+/+	20.4±2.1	23.9±4.3	29.6±3.2**	19.2±2.3
	MT−/−	21.8±1.7	18.7±2.0* #	38.5±2.9** ##	26.0±2.0** ##
GPx (U/mg pro)	MT+/+	37.9±4.0	33.6±2.2*	20.4±3.3**	25.5±4.1**
	MT−/−	41.1±2.9	36.3±3.3*	21.6±4.7**	26.1±2.9**
GR (nmol/min/mg pro)	MT+/+	8.3±1.7	7.2±1.5	17.5±2.8**	11.1±1.0*
	MT−/−	9.2±1.0	7.4±1.7	21.1±2.0** #	16.6±1.4** ##
SOD (U/mg pro)	MT+/+	151.6±9.5	163.7±7.2*	149.6±5.3	150.3±10.6
	MT−/−	136.0±5.2##	132.9±8.4##	139.7±6.7#	136.0±5.8##

** p<0.01, compared with respective control;

* p<0.05, compared with respective control;

## p<0.01, compared with MT+/+ mice for the same treatment;

# p<0.05, compared with MT+/+ mice for the same treatment.

### Protein Expression of CYP2E1 and CYP1A2 in Wild-type and MT−/− Mice

**Figure 4:**
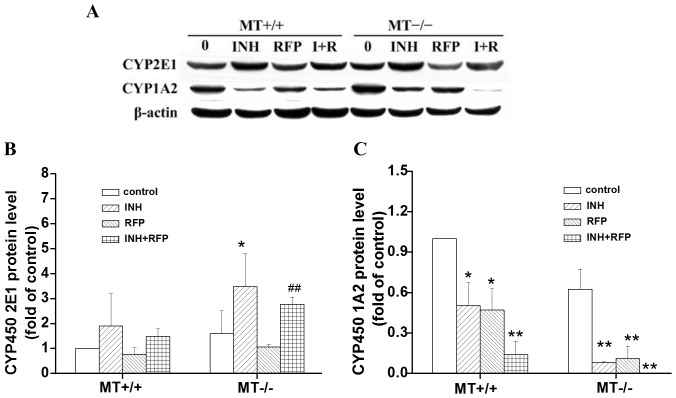
Effect of INH and RFP treatment on the expressions of CYP2E1 and CYP1A2 in wild-type (MT^+/+^) and MT−/− mice. The expression of CYP2E1 and CYP1A2 was analyzed by Western blotting. **p<0.01, compared with respective control; *p<0.05, compared with respective control; ^##^p<0.01, compared with MT^+/+^ mice for the same treatment.

Previous evidence suggests that cytochrome P450s (CYPs) are associated with the development of INH and RFP induced hepatotoxicity, particularly CYP2E1 and CYP1A2 [32], [33]. In western blot analysis (Fig. 4), the exposure to INH alone or the combined exposure significantly upregulated hepatic CYP2E1 protein expression, and the induction in the MT−/− mice was more significant. We observed that exposure to INH and RFP alone or in combination significantly inhibited hepatic CYP1A2 protein expression.

## Discussion

The administration of INH and RFP produces many metabolic and morphological aberrations in the liver due to the fact that the liver is the main detoxifying site for these antitubercular drugs [11]. Increasing evidence suggests that these toxic metabolites can induce oxidative stress in the liver of experimental rats [34], [35]. Metushi et al. found that INH itself can be oxidized to a reactive metabolite that covalently binds to mouse and human hepatic proteins, and INH-induced liver injury is immune-mediated [36]. An antioxidant role for MT has been proposed based on the observations that animals that overexpress MT due to chemical induction or to direct gene transfer are resistant to several forms of oxidative injuries, and animals that are deficient in MT levels due to gene knockout experience enhanced sensitivity to oxidative injury [37]–[39]. In the present study, we used MT-I/II null (MT−/−) mice to reveal the protective role of basal expression of MT against INH and RFP-induced hepatic oxidative stress. We found that INH and RFP treatment induced more significant hepatic histopathological injuries, increases of MDA production, serum AST level and liver index in MT−/− mice than in wild-type mice, indicating the inability to produce MT seems to be related to the enhanced susceptibility of MT−/− mice to hepatic oxidative stress. Studies have demonstrated that MT acts as a scavenger and can interact directly with oxygen free radicals [40], and inhibits these toxic radicals inducing lipid, protein and DNA damage [41]–[43].

In biological systems, the balance between pro-oxidants and antioxidants is crucial to cellular homeostasis. As observed in the study, INH and RFP caused the impairment of liver antioxidant status, as evidenced by the significant decrease in the activity of GPx, and increases in the level of GSH and the activity of GR, especially in MT−/− mice. As is well known, cells have evolved an array of well-coordinated defense mechanisms comprising antioxidant molecules such as GSH, and antioxidant enzymes SOD, GPx and GR, all of which act synergistically to detoxify the oxidative injury by means of scavenging oxygen free radicals. GSH is one of the most prominent antioxidant defense components in the liver. Besides serving as a substrate for glutathione-related enzymes such GPx, GSH acts as a free radical scavenger, and plays an important role in the maintenance of protein sulfhydryls. GR is able to catalyse the reduction of the oxidized form of glutathione. MT shares an important similarity with GSH due to the fact that one-third of its amino acids are cysteines, and both MT and GSH are the main sources of sulfhydryls in the liver [25]. Importantly, the sulfhydryls in MT are preferential targets of free radical attack compared with the other sulfhydryls such as those from GSH and protein fractions [44]. It is reasonable to consider that the anti-oxidative capacity of the MT−/− mice is reinforced partly by the compensatory increase of GSH and GR because of MT deficiency. We also find the basal level of SOD-1 protein in MT−/− mice was significantly lower than that in wild-type mice. It can be inferred as the maintaince of SOD-1 in MT+/+ mice helps ameliorate toxicity, and MT may also play a role in stabilizing the total anti-oxidative capacity in the liver of the MT+/+ mice.

Although INH and RFP are potentially hepatotoxic drugs, the precise molecular mechanisms behind their adverse effects in the body are not fully understood. INH is metabolized to acetylisoniazid via hepatic N-acetyltransferase2 [45]. In turn, acetylisoniazid is hydrolyzed to acetylhydrazine, which is oxidized by cytochrome P450 to form some hepatotoxic intermediates [46], [47]. Cytochrome P450 enzymes (CYPs) are involved in the metabolism of endogenous substances, drugs, hormones, and xenobiotics. There is ample evidence that elevation of hepatic CYP2E1 plays an essential role in the INH-hepatotoxicity through generation of free radicals from hydrazine [48], [49]. The cytotoxicity mediated by CYP 2E1 has been found to be closely related to its oxy-radical producing ability, leading to lipid peroxidation [50]. Additionally, cytochrome P450 are thought to contribute to the additive or synergistic effects of RFP on INH-induced hepatotoxicity [51], [52]. In addition to the activation of CYP2E1, an inhibiting effect on CYP1A2 was considered another molecular mechanism of the INH-toxicity [53]. Our studies showed the significant induction of CYP2E1 protein expression and inhibition of CYP1A2 protein expression after INH treatment in the MT−/− mice, indicating that MT−/− mice were more susceptible than MT+/+ mice to oxidative injury mediated by cytochrome P450. RFP alone or in combination with INH significantly inhibited CYP1A2 protein expression, and this effect was exaggerated in the MT−/− mice. These findings clearly demonstrate that basal MT provides protection against INH and RFP-induced toxicity in hepatocytes. The CYP2E1 and CYP1A2 were involved in the pathogenesis of INH and RFP-induced hepatotoxicity.

Several studies have proven that RFP increases INH toxicity, most probably by increasing the formation of its toxic metabolite hydrazine [54], [55]. However, a low rate of hepatotoxicity was demonstrated after INH-RFP co-administration for 12 weeks, which was similar to INH treatment alone in a Hong Kong study [56]. It has further been reported that RFP co-administration does not increase INH-induced oxidative stress through hepatic CYP2E1 during short-term treatment in experimental rats [57]. In the present study, the results from biochemistry analysis and histopathological examinations indicated INH–RFP co-administration did not exacerbate liver damage in mice. RFP attenuated INH-induced CYP2E1 protein expression, indicating RFP may not exacerbate INH-induced free radical generation, and our results are consistent with previous findings that RFP reduced hepatic CYP2E1 and had a protective effect on liver injury induced by carbon tetrachloride [58], [59]. In a recent paper, Shen et al. provided interesting findings that RFP exacerbated INH toxicity in human hepatocytes but not in rat hepatocytes because of the difference in induction of CYP 2E1. So when comparing data on drug toxicity between species, particular attention is need to pay on drug-metabolizing enzymes [60].

In summary, the present study reveals that MT-I/II null mice are more sensitive than wild type mice to the toxic effects of INH and RFP, confirming basal expression of MT provides protection against the hepatotoxicity. The CYP2E1 and CYP1A2 were involved in the pathogenesis of INH and RFP-induced hepatotoxicity. As a phase II detoxifying enzymes, MT contain specific nucleotide sequences in the gene promoters that contribute to the protection of cells against oxidative stress [61]. In recent years, NF-E2-related factor 2 (Nrf2) has been studied by many scientists and was considered can mediate a multitude of antioxidant signaling and detoxification genes [62], [63]. Several reports have suggested a protective action for Nrf2-ARE signalling pathway against CYP2E1-dependent hepatic oxidative injury [64], [65], indicating Nrf2-ARE pathway may exhibit a protective effect on INH and RFP-induced hepatotoxicity. In addition, Weng et al. showed expression of MT may function by activating the phosphorylation of JNK, p38 and PI3K/Akt as well as by enhancing Nrf2 DNA-binding activity [66]. Therefore, more studies of relation between MT and Nrf2-ARE pathway, and the actual mechanism underlying these activities are needed for the elucidation of the hepatoprotective mechanisms of MT.
